# Investigation of the association of serum GFAP and NfL with brain and upper cervical MRI volumes in AQP4-IgG-positive NMOSD and MOGAD

**DOI:** 10.1177/17562864251345792

**Published:** 2025-07-20

**Authors:** Patrick Schindler, Ulrike Grittner, Rebekka Rust, Susanna Asseyer, Judith Bellmann-Strobl, Tanja Schmitz-Hübsch, Michael Scheel, Sven Jarius, Brigitte Wildemann, Markus Reindl, Pascal Benkert, Jens Kuhle, Friedemann Paul, Klemens Ruprecht, Claudia Chien

**Affiliations:** Department of Neurology, Charité—Universitätsmedizin Berlin, corporate member of Freie Universität Berlin and Humboldt-Universität zu Berlin, Berlin, Germany; Experimental and Clinical Research Center, a cooperation between the Max Delbrück Center for Molecular Medicine in the Helmholtz Association and the Charité—Universitätsmedizin Berlin, Berlin, Germany; Neuroscience Clinical Research Center, Charité—Universitätsmedizin Berlin, corporate member of Freie Universität Berlin and Humboldt-Universität zu Berlin, and Berlin Institute of Health, Berlin, Germany; Institute of Biometry and Clinical Epidemiology, Charité—Universitätsmedizin Berlin, Corporate Member of Freie Universität Berlin and Humboldt-Universität zu Berlin, Berlin, Germany; Experimental and Clinical Research Center, a cooperation between the Max Delbrück Center for Molecular Medicine in the Helmholtz Association and the Charité—Universitätsmedizin Berlin, Berlin, Germany; Neuroscience Clinical Research Center, Charité—Universitätsmedizin Berlin, Corporate Member of Freie Universität Berlin and Humboldt-Universität zu Berlin, and Berlin Institute of Health, Berlin, Germany; Institute of Medical Immunology, Charité—Universitätsmedizin Berlin, Corporate Member of Freie Universität Berlin and Humboldt-Universität zu Berlin, Berlin, Germany; Department of Neurology, Charité—Universitätsmedizin Berlin, corporate member of Freie Universität Berlin and Humboldt-Universität zu Berlin, Berlin, Germany; Experimental and Clinical Research Center, a cooperation between the Max Delbrück Center for Molecular Medicine in the Helmholtz Association and the Charité—Universitätsmedizin Berlin, Berlin, Germany; Neuroscience Clinical Research Center, Charité—Universitätsmedizin Berlin, corporate member of Freie Universität Berlin and Humboldt-Universität zu Berlin, and Berlin Institute of Health, Berlin, Germany; Experimental and Clinical Research Center, a cooperation between the Max Delbrück Center for Molecular Medicine in the Helmholtz Association and the Charité—Universitätsmedizin Berlin, Berlin, Germany; Neuroscience Clinical Research Center, Charité—Universitätsmedizin Berlin, Corporate Member of Freie Universität Berlin and Humboldt-Universität zu Berlin, and Berlin Institute of Health, Berlin, Germany; Experimental and Clinical Research Center, a cooperation between the Max Delbrück Center for Molecular Medicine in the Helmholtz Association and the Charité—Universitätsmedizin Berlin, Berlin, Germany; Neuroscience Clinical Research Center, Charité—Universitätsmedizin Berlin, Corporate Member of Freie Universität Berlin and Humboldt-Universität zu Berlin, and Berlin Institute of Health, Berlin, Germany; Department of Neuroradiology, Charité—Universitätsmedizin Berlin, Corporate Member of Freie Universität Berlin and Humboldt-Universität zu Berlin, Berlin, Germany; Molecular Neuroimmunology Group, Department of Neurology, University of Heidelberg, Heidelberg, Germany; Molecular Neuroimmunology Group, Department of Neurology, University of Heidelberg, Heidelberg, Germany; Clinical Department of Neurology, Medical University of Innsbruck, Innsbruck, Austria; Department of Clinical Research, University Hospital Basel, University of Basel, Basel, Switzerland; MS Center and Research Center for Clinical Neuroimmunology and Neuroscience Basel (RC2NB), University Hospital Basel, Neurology Clinic and Policlinic, Basel, Switzerland; Department of Neurology, Charité—Universitätsmedizin Berlin, corporate member of Freie Universität Berlin and Humboldt-Universität zu Berlin, Berlin, Germany; Experimental and Clinical Research Center, a cooperation between the Max Delbrück Center for Molecular Medicine in the Helmholtz Association and the Charité—Universitätsmedizin Berlin, Berlin, Germany; Neuroscience Clinical Research Center, Charité—Universitätsmedizin Berlin, corporate member of Freie Universität Berlin and Humboldt-Universität zu Berlin, and Berlin Institute of Health, Berlin, Germany; Department of Neurology, Charité—Universitätsmedizin Berlin, corporate member of Freie Universität Berlin and Humboldt-Universität zu Berlin, Berlin, Germany; Experimental and Clinical Research Center, a cooperation between the Max Delbrück Center for Molecular Medicine in the Helmholtz Association and the Charité—Universitätsmedizin Berlin, Berlin, Germany; Neuroscience Clinical Research Center, Charité—Universitätsmedizin Berlin, corporate member of Freie Universität Berlin and Humboldt-Universität zu Berlin, and Berlin Institute of Health, Berlin, Germany; Department of Psychiatry and Neurosciences, Charité—Universitätsmedizin Berlin, corporate member of Freie Universität Berlin and Humboldt-Universität zu Berlin, Berlin, Germany

**Keywords:** biomarker, glial fibrillary acidic protein (GFAP), magnetic resonance imaging (MRI), myelin oligodendrocyte glycoprotein antibody-associated disease (MOGAD), neurofilament light chain (NfL), neuromyelitis optica spectrum disorders (NMOSD)

## Abstract

**Background:**

Serum glial fibrillary acidic protein (sGFAP) is associated with disease activity in aquaporin-4-immunoglobulin G-seropositive neuromyelitis optica spectrum disorders (AQP4-IgG+NMOSD). Serum neurofilament light chain (sNfL) is a biomarker for neuroaxonal damage. However, the association of sGFAP and sNfL with magnetic resonance imaging (MRI) volumes in AQP4-IgG+NMOSD is unclear.

**Objectives:**

To investigate the associations of sGFAP and sNfL with brain MRI volumes in AQP4-IgG+NMOSD.

**Design:**

Monocentric, retrospective, observational study.

**Methods:**

In 33 clinically stable patients with AQP4-IgG+NMOSD, 17 patients with myelin oligodendrocyte glycoprotein antibody-associated disease (MOGAD), and 15 healthy controls (HC), sGFAP and sNfL were measured at 2 (HC = 1) and 3-Tesla MRIs were obtained at 4 (HC = 1) yearly visits. Associations between biomarkers and MRI metrics were evaluated using linear models.

**Results:**

In AQP4-IgG+NMOSD, but not in MOGAD and HC, higher sGFAP was associated with lower hippocampus (β = −2.0 (95% confidence interval: −3.4, −0.7), *p* = 0.004) and thalamus volumes (β = −2.5 (−4.3, −0.7), *p* = 0.006) and higher MRI cerebrospinal fluid volume (β = 1.8 (0.7, 3.2), *p* = 0.01), and, statistically less robust, with lower whole brain (β = −2.3 (−5.3, 0.8), *p* = 0.15) and gray matter volumes (β = −1.8 (−4.0, 0.4), *p* = 0.10). Furthermore, higher sGFAP (β = −0.06 (−0.11, −0.002), *p* = 0.04), but not sNfL (β = −0.02 (−0.08, 0.03), *p* = 0.38), was associated with percent brain volume change in AQP4-IgG+NMOSD.

**Conclusion:**

The specific associations of sGFAP with brain MRI volumes corroborate sGFAP as a biomarker for disease activity in AQP4-IgG+NMOSD.

## Introduction

Aquaporin-4-immunoglobulin G-seropositive neuromyelitis optica spectrum disorder (AQP4-IgG+NMOSD) is a relapsing autoimmune astrocytopathy.^1,2^ Although treatment options for attack prevention in AQP4-IgG+NMOSD have rapidly expanded in recent years, biomarkers for monitoring disease activity are still insufficiently established and thus represent an important unmet need.

Magnetic resonance imaging (MRI) is paramount for the differential diagnosis of neuromyelitis optica spectrum disorders (NMOSD).^1^ Furthermore, MRI volumetry is increasingly investigated in NMOSD^3^ and demonstrated that patients with NMOSD have reduced white matter^4^ and specific gray matter structural volumes compared to healthy controls (HC).^5–7^ Specifically, thalamic atrophy has consistently been detected in patients with NMOSD,^5,8^ and thalamic and hippocampal atrophy have been linked to cognitive impairment in NMOSD.^8,9^

Glial fibrillary acidic protein (GFAP) is an intermediate filament abundant in astrocytes.^10^ Accumulating evidence suggests that serum GFAP (sGFAP) represents a biomarker for disability and disease activity in AQP4-IgG+NMOSD, reflecting disease-specific end-organ damage.^10^ Additionally, sGFAP may be predictive for attack risk in AQP4-IgG+NMOSD.^11,12^ sGFAP levels are associated with complementary biomarker candidates in AQP4-IgG+NMOSD, including serum neurofilament light chain (sNfL),^12^ a marker for neuroaxonal injury, and retinal nerve fiber layer thickness.^13^

However, while limited evidence suggests an association of higher sGFAP with spinal and cerebral lesions in AQP4-IgG+NMOSD,^11,14,15^ systematic investigations of the association of sGFAP with MRI volumes in patients with NMOSD have so far not been conducted. Therefore, it remains unclear whether structural damage detected by MRI in patients with AQP4-IgG+NMOSD may be reflected by sGFAP.

Here, we performed an explorative investigation of cross-sectional and longitudinal associations between brain MRI volumes and sGFAP in clinically stable patients with AQP4-IgG+NMOSD. In addition, we analyzed the association of MRI volumes and sNfL. To evaluate the specificity of our findings, we also included patients with myelin oligodendrocyte glycoprotein antibody-associated disease (MOGAD) and HC.

## Participants and methods

### Participants and study design

This is a monocentric, retrospective, observational investigation. Thirty-three patients with AQP4-IgG+NMOSD and 17 with MOGAD diagnosed according to current criteria,^1,16,17^ were included in a prospective longitudinal observational study. Inclusion criteria for the present analysis were age of at least 18 years at inclusion and ability to give informed consent. Exclusion criteria were contraindications or inability to undergo MRI and optical coherence tomography (Supplemental Figure 1). Additionally, 15 HC were included. No sample size calculation was performed, as all available persons fulfilling the criteria were included. Participants of this study were previously reported in studies on either sGFAP and sNfL or MRI in NMOSD, MOGAD, and HC, as described in detail in the Supplemental Material. All patients with AQP4-IgG+NMOSD and MOGAD enrolled in the abovementioned prospective observational study at the time of sGFAP and sNfL measurement and with available serum samples and MRI scans at the respective time point were included; therefore, no further inclusion or exclusion criteria were applied (Supplemental Figure 1). HCs were selected to match as close as possible the age and sex distribution of patients on a cohort level.

**Figure 1.**
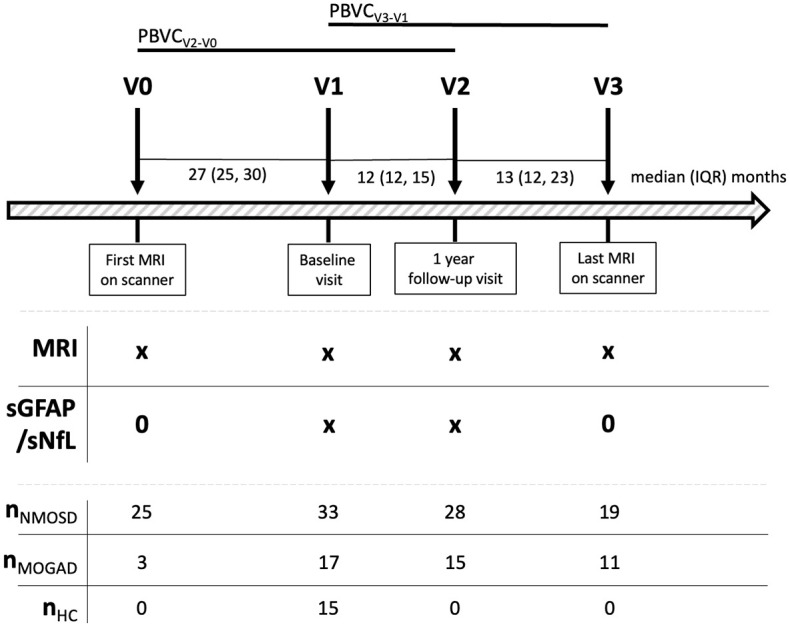
Study design. Arrows indicate study visits (V) at the respective time point (0/1/2/3). Detailed clinical data were obtained at V1, V2, and V3. AQP4-IgG+NMOSD, aquaporin-4-immunoglobulin G-seropositive neuromyelitis optica spectrum disorder; HC, healthy controls; IQR, interquartile range; MOGAD, myelin oligodendrocyte glycoprotein antibody-associated disease; MRI, magnetic resonance imaging; NMOSD, neuromyelitis optica spectrum disorders; *n*NMOSD/MOGAD/HC, number of AQP4-IgG+NMOSD patients/MOGAD patients/HC; PBVC, percent brain volume change; sGFAP, serum glial fibrillary acidic protein; sNfL, serum neurofilament light chain; V, visits.

The study design is depicted in Figure 1. Identical procedures were applied at each study visit to obtain serum samples and MRI scans. All patients (NMOSD and MOGAD) were in clinical remission (as defined by a time interval >30 days since onset of last attack) at visit 1 (V1) and visit 2 (V2).

### Methods

#### Laboratory procedures

Serum AQP4-IgG and MOG-IgG were determined using fixed or live cell-based assays.^18^ sGFAP and sNfL concentrations were measured by Simoa, as previously described.^14^

#### Magnetic resonance imaging

All 3D T1- and T2-weighted cerebral MRI scans were obtained as previously reported^6^ on a 3-Tesla Siemens Trio scanner, with T2-hyperintense lesion segmentation manually performed by two expert MRI-technicians (>10 years of experience) on T2-weighted fluid attenuated inversion recovery scans. Volumes of brain structures and cranial MRI cerebrospinal fluid (CSF) volume were determined based on lesion-filled T1-weighted magnetization-prepared rapid gradient echo scans by application of FSL SIENAX^19^ and FSL FIRST^20^ and then multiplied with the SIENAX V-Scaling factor for normalization. As a measure of longitudinal brain atrophy, we calculated percent brain volume change (PBVC) using FSL SIENA.^19^ To differentiate between past and future brain atrophy relative to the time of serum sampling, we calculated PBVC_V2 − V0_ between the first available brain MRI scan (V0, or V1 if V0 was not available) of each patient and the scan at V2 as well as PBVC_V3 − V1_ between the scan at V1 and the last available scan (V3) of each patient (Figure 1).

#### Statistical analyses

Log-transformation was performed for sGFAP and sNfL, and rank-transformation for delta sGFAP and delta sNfL, enabling the use of parametric tests. For intergroup differences, standardized mean differences (SMD) were calculated. For descriptive group comparisons, unadjusted *p*-values were derived from Chi-square test, one-way-ANOVA, and Kruskal–Wallis test, as applicable. Cross-sectional associations between sGFAP or sNfL and MRI measures were analyzed using multivariable linear models adjusted for age and sex and including an interaction term of diagnosis with MRI measure. For longitudinal analyses, similar linear models were calculated, with delta sGFAP/sNfL (V2 − V1) as independent or PBVC as dependent variables. Marginal effect estimates beta (β) were calculated (package “emmeans”). For models, all MRI parameters were rescaled by division through the respective maximum. Standardized effect sizes (SES) were calculated for interaction effects (package “effectsize”). Due to the exploratory design of this study, interpretations are based on effect sizes (β, SMD, SES) and no correction for multiple testing was applied. Accordingly, all *p*-values should be interpreted cautiously. As per inclusion criteria, there were no missing sGFAP/sNfL data at V1. For longitudinal analyses, only complete datasets were used, no imputation of missing values was conducted. All statistical analyses were performed using R (version 4.2.2).^21^

Further details of the study design, study participants, laboratory procedures, MRI, and statistical analyses are provided in the Supplemental Material.

## Results

### Study participants

Baseline demographic and clinical characteristics of the study participants are summarized in Table 1. Patients with AQP4-IgG+NMOSD were older and more often female than HC and patients with MOGAD (Table 1). Disease duration and time since the last attack at V1 were longer, and the expanded disability status scale score was higher in patients with AQP4-IgG+NMOSD compared to MOGAD (Table 1). The frequency of the cardiovascular risk factors diabetes and hypertension was similar in AQP4-IgG+NMOSD and MOGAD, while smoking was more prevalent in MOGAD (Table 1). Six of 28 (21.4%) AQP4-IgG+NMOSD patients and 5 of 15 (33%, SMD = 0.27, *p* = 0.27) MOGAD patients with 1 year follow-up had an attack during this period (V1 − V2), no patient had more than one attack.

**Table 1.** Baseline demographic and clinical characteristics.

Demographic and clinical characteristics	NMOSD	MOGAD	HC	SMD			*p*		
				NMOSD vs MOGAD	NMOSD vs HC	MOGAD vs HC	NMOSD vs MOGAD	NMOSD vs HC	MOGAD vs HC
*N*	33	17	15						
Age (years), median (IQR)	50 (40, 59)	48 (34, 55)	36 (32, 60)	0.26	0.40	0.14	0.37	0.16	0.71
Female (%)	30 (90.9)	11 (64.7)	12 (80.0)	0.66	0.31	0.35	0.06	0.56	0.57
Ever smoked (%)	15 (45.5)	12 (75.0)		0.63			0.10		
Arterial hypertension (%)	7 (21.2)	4 (23.5)		0.06			1.00		
Diabetes mellitus (%)	0 (0)	0 (0)							
Months since last attack, median (IQR)	31 (12, 56)	6 (5, 19)		0.63			0.002		
Disease duration (months), median (IQR)	76 (52, 96)	43 (10, 132)		0.07			0.13		
EDSS, median (IQR)	4.0 (2.0, 4.5)	2.5 (2.0, 3.0)		0.66			0.04		
Current IMT (%)	29 (87.9)	13 (76.5)		0.30			0.53		

*p*-Values are unadjusted, derived from Chi-square test for categorical variables, from one-way-ANOVA for continuous normally distributed variables, and from Kruskal–Wallis test for continuous nonnormally distributed variables.

CSF, cerebrospinal fluid; EDSS, expanded disability status scale; HC, healthy controls; IMT, immunotherapy; IQR, interquartile range; MOGAD, myelin oligodendrocyte glycoprotein antibody-associated disease; NMOSD, neuromyelitis optica spectrum disorder; sGFAP, serum glial fibrillary acidic protein; SMD, standardized mean difference; sNfL, serum neurofilament light chain; vol., volume.

sGFAP and sNfL concentrations as well as MRI volumes are shown in Table 2. Patients with AQP4-IgG+NMOSD had a higher median baseline sGFAP concentration (109.2 pg/ml) than HC (66.4 pg/ml, SMD = 0.8, *p* = 0.03) and, to a lesser degree, than patients with MOGAD (77.7 pg/ml, SMD = 0.1, *p* = 0.31). sNfL concentrations were similar between all three groups (Table 2). The median intraindividual percentual change, that is the intraindividual variability, of sGFAP between baseline and V2 was similar in AQP4-IgG+NMOSD (17% (IQR 7%–22%)) and MOGAD (16% (12%–18%), SMD = 0.3, *p* = 0.80), as was the median intraindividual percentual change of sNfL (AQP4-IgG+NMOSD: 24% (9%–33%), MOGAD: 17% (10%–35%), SMD = 0.4, *p* = 1.00). Baseline brain volume (SMD = 0.8, *p* = 0.01), gray matter volume (SMD = 0.7, *p* = 0.04), and pallidal volume (SMD = 0.8, *p* = 0.03) were lower, and thalamic (SMD = 0.6, *p* = 0.06) and white matter (SMD = 0.6, *p* = 0.07) volumes were nonsignificantly lower in AQP4-IgG+NMOSD than in HCs. Moreover, amygdala volume was lower in AQP4-IgG+NMOSD than in MOGAD (SMD = 0.8, *p* = 0.007). Mean upper cervical spinal cord area was lower in patients with AQP4-IgG+NMOSD than in HCs (SMD = 0.9, *p* = 0.008), and nonsignificantly lower in AQP4-IgG+NMOSD than in MOGAD (SMD = 0.5, *p* = 0.08). The cerebral T2-lesion count was higher in patients with AQP4-IgG+NMOSD compared to HCs (SMD = 0.4, *p* = 0.02) and patients with MOGAD (SMD = 0.7, *p* < 0.001).

**Table 2.** Serum biomarker concentrations and brain MRI volumes.

Serum and MRI measurements	NMOSD	MOGAD	HC	SMD			*p*		
				NMOSD vs MOGAD	NMOSD vs HC	MOGAD vs HC	NMOSD vs MOGAD	NMOSD vs HC	MOGAD vs HC
*N*	33	17	15						
Serum biomarkers									
sGFAP (pg/ml), median (IQR)	109.2 (69.8, 159.4)	77.7 (61.6, 104.7)	66.4 (56.2, 97.2)	0.08	0.80	0.49	0.31	0.03	0.22
sNfL (pg/ml), median (IQR)	22.3 (17.1, 38.9)	25.3 (19.4, 43.6)	24.1 (17.8, 34.6)	0.18	0.08	0.25	0.59	0.96	0.64
Brain MRI volumetry									
Brain vol. (ml), mean (SD)	1491.9 (77.4)	1525.8 (102.3)	1552.2 (69.3)	0.37	0.82	0.30	0.20	0.01	0.41
Gray matter vol. (ml), mean (SD)	781.6 (63.9)	803.9 (78.2)	821.8 (56.6)	0.31	0.67	0.26	0.29	0.04	0.47
White matter vol. (ml), mean (SD)	710.3 (33.9)	721.9 (40.4)	730.4 (36.1)	0.31	0.57	0.22	0.29	0.07	0.54
CSF vol. (ml), mean (SD)	486.7 (46.3)	481.8 (59.8)	465.1 (47.8)	0.09	0.46	0.31	0.75	0.14	0.39
Thalamus vol. (ml), mean (SD)	20.4 (1.7)	20.7 (2.2)	21.4 (1.7)	0.12	0.61	0.40	0.67	0.06	0.27
Caudatus vol. (ml), mean (SD)	9.0 (1.06)	9.2 (1.1)	9.6 (1.0)	0.22	0.55	0.30	0.46	0.09	0.40
Putamen vol. (ml), mean (SD)	12.6 (1.4)	12.8 (1.0)	13.2 (0.9)	0.19	0.52	0.39	0.55	0.12	0.29
Pallidum vol. (ml), mean (SD)	4.5 (0.4)	4.5 (0.5)	4.8 (0.3)	0.01	0.77	0.70	0.96	0.03	0.06
Hippocampus vol. (ml), mean (SD)	10.2 (1.1)	10.0 (1.7)	10.5 (0.8)	0.1	0.263	0.36	0.59	0.43	0.33
Amygdala vol. (ml), mean (SD)	3.8 (0.5)	3.4 (0.5)	3.6 (0.6)	0.84	0.42	0.36	0.007	0.17	0.32
Accumbens vol. (ml), mean (SD)	1.1 (0.2)	1.1 (0.3)	1.2 (0.2)	0.002	0.20	0.18	1.00	0.54	0.62
Brainstem vol. (ml), mean (SD)	29.6 (2.4)	29.0 (2.8)	29.4 (1.8)	0.23	0.09	0.17	0.44	0.79	0.64
MUCCA (mm^2^), mean (SD)	67.2 (6.6)	72.0 (12.2)	72.7 (5.5)	0.49	0.90	0.07	0.08	0.008	0.84
Brain T2-lesion count, median (IQR)	17 (10, 38)	4 (1, 7)	4 (3, 25)	0.68	0.40	0.31	<0.001	0.02	0.47
Brain T2-lesion vol. (ml), median (IQR)	1.1 (0.5, 1.9)	0.1 (0.05, 0.6)	0.2 (0.06, 0.4)	0.07	0.74	0.30	<0.001	0.001	0.66
Longitudinal brain atrophy									
PBVC_V2 − V0_, mean (SD)	*n* = 22	*n* = 12		0.50			0.17		
	−1.1 (0.8)	−0.7 (0.8)							
PBVC_V3 − V1_, mean (SD)	*n* = 19	*n* = 10		0.21			0.60		
	−0.9 (1.0)	−0.7 (0.9)							

All cross-sectional measures refer to data obtained at baseline (V1). *p*-Values are unadjusted, derived from Chi-square test for categorical variables, from one-way-ANOVA for continuous normally distributed variables, and from Kruskal–Wallis test for continuous nonnormally distributed variables. Longitudinal brain atrophy was assessed in patients with complete (i.e., sGFAP and sNfL at V1 and V2, and PBVC) longitudinal datasets.

CSF, cerebrospinal fluid; HC, healthy controls; IMT, immunotherapy; IQR, interquartile range; MOGAD, myelin oligodendrocyte glycoprotein antibody-associated disease; MRI, magnetic resonance imaging; MUCCA, mean upper cervical spinal cord area; NMOSD, neuromyelitis optica spectrum disorder; PBVC, percent brain volume change; SD, standard deviation; sGFAP, serum glial fibrillary acidic protein; SMD, standardized mean difference; sNfL, serum neurofilament light chain; V, visits; vol., volume.

Longitudinal brain atrophy, as assessed by PBVC, did not substantially differ between patients with AQP4-IgG+NMOSD and MOGAD (Table 2).

### Baseline associations between sGFAP, sNfL, and structural brain MRI measures

**Figure 2.**
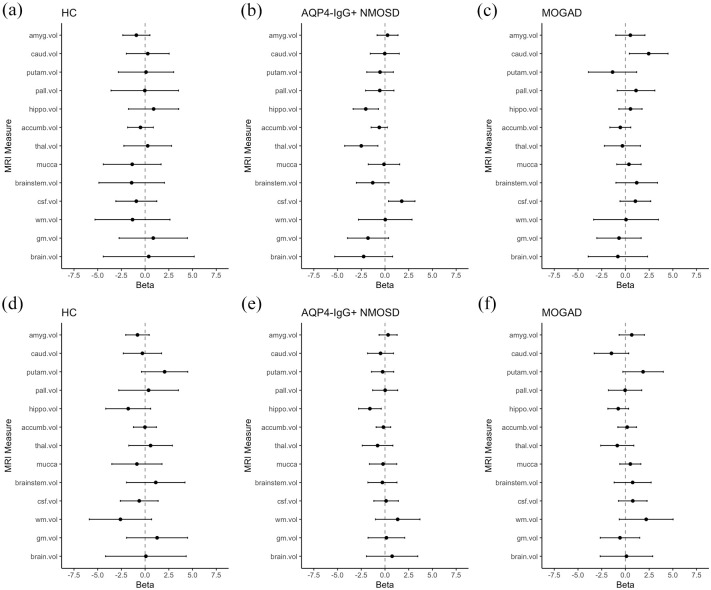
Effect sizes of baseline associations of sGFAP (a–c) and sNfL (d–f) with brain MRI structural measuresDots indicate the comparable coefficient β, derived from linear models, one model per MRI parameter, adjusted for age and sex and with an interaction term of MRI parameter and diagnosis. Horizontal lines indicate the corresponding 95% confidence interval. One model was calculated for each MRI parameter, each including all three groups. sGFAP or sNfL were used as dependent and MRI parameter as independent variables. Number of included individuals: NMOSD *n* = 33, MOGAD *n* = 17, HC *n* = 15. Accumb.vol, accumbens volume; amyg.vol, amygdala volume; AQP4-IgG+NMOSD, aquaporin 4-immunoglobulin G-seropositive neuromyelitis optica spectrum disorder; brain.vol, brain volume; brainstem.vol, brainstem volume; caud.vol, caudate volume; csf.vol, cranial MRI cerebrospinal fluid volume; gm.vol, gray matter volume; HC, healthy controls; hippo.vol, hippocampus volume; MOGAD, myelin oligodendrocyte glycoprotein antibody-associated disease; MRI, magnetic resonance imaging; MUCCA, mean upper cervical spinal cord area; NMOSD, neuromyelitis optica spectrum disorders; putam.vol, putaminal volume; sGFAP, serum glial fibrillary acidic protein; sNfL, serum neurofilament light chain; thal.vol, thalamus volume; wm.vol, white matter volume.

We analyzed the association of sGFAP and sNfL with structural brain MRI measures in patients with AQP4-IgG+NMOSD, MOGAD, and HC at the baseline visit (V1) by calculating comparable coefficients (β) adjusted for age and sex (Figure 2). Corresponding scatterplots are provided in Supplemental Figure 2.

In AQP4-IgG+NMOSD, higher sGFAP was associated with lower hippocampus volume (β = −2.0 (95% confidence interval: −3.4, −0.7), *p* = 0.004), lower thalamus volume (β = −2.5 (−4.3, −0.7), *p* = 0.006), and higher cranial MRI CSF volume (β = 1.8 (0.7, 3.2), *p* = 0.01). A statistically less robust association was detected between higher sGFAP and lower whole brain volume (β = −2.3 (−5.3, 0.8), *p* = 0.15), which was driven by a nonsignificant association of higher sGFAP with lower gray matter volume (β = −1.8 (−4.0, 0.4), *p* = 0.10), but not white matter volume (β = 0.03 (−2.8, 2.9), *p* = 0.98).

In patients with MOGAD, the associations with sGFAP observed in AQP4-IgG+NMOSD were either less pronounced or absent (Figure 2). The difference between AQP4-IgG+NMOSD and MOGAD was strongest regarding the associations of sGFAP with hippocampus (SES_interaction (int)_ = 0.7 (0.3, 1.2), *p*_int_ = 0.002) and thalamus volume (SES_int_ = 0.5 (−0.03, 1.0), *p*_int_ = 0.07). Of note, higher caudate volume was associated with higher sGFAP in MOGAD (β = 2.5 (0.4, 4.5), *p* = 0.02), but not in AQP4-IgG+NMOSD (β = −0.02 (−1.5, 1.5), **p* =* 0.97, SES_int_ = −0.6 (−1.1, −0.05), *p*_int_ = 0.03).

Likewise, the associations between sGFAP and structural brain measures were either less pronounced or absent in HC. Again, interaction effects by group were detected for the association of sGFAP with hippocampus volume (SES_int_ = 0.8 (0.04, 1.6), *p*_int_ = 0.04) and with thalamus volume (SES_int_ = 0.6 (−0.01, 1.2), *p*_int_ = 0.06).

Higher sNfL was associated with lower hippocampus volume in AQP4-IgG+NMOSD (β = −1.6 (−2.8, −0.4), *p* = 0.01), and, statistically less robust, in MOGAD (β = −0.8 (−1.9, 0.3), *p* = 0.17, SES_int_ = 0.2 (−0.2, 0.6), *p*_int_ = 0.25) and HC (β = −1.8 (−4.2, 0.6), *p* = 0.14, SES_int_ = −0.05 (−0.7, 0.6), *p*_int_ = 0.88). No clear further associations between sNfL and MRI parameters were observed.

### Association of sGFAP and sNfL with longitudinal brain atrophy

To assess the association of sGFAP and sNFL with longitudinal brain atrophy, we analyzed PBVC in the subset of patients with complete longitudinal datasets, that is PBVC, longitudinal sGFAP, and longitudinal sNfL data. The median interval for determination of PBVC_V2 − V0_ was 39 (IQR 28–41) months for AQP4-IgG+NMOSD (*n* = 22) and 12 (12–20) months for MOGAD (*n* = 12). For PBVC_V3 − V1_, the median interval was 26 (25–36) months for AQP4-IgG+NMOSD (*n* = 19) and 25 (24–26) months for MOGAD (*n* = 10). We investigated the association (1) of change in sGFAP and sNfL levels with brain atrophy, (2) of sGFAP and sNfL at a given point of time with prior brain atrophy, and (3) of sGFAP and sNfL at a given point of time with future brain atrophy.

**Figure 3.**
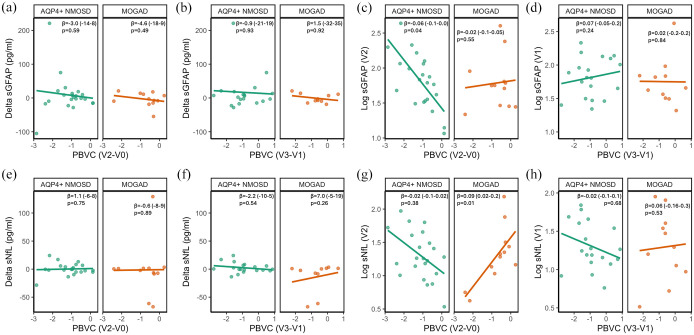
Association of change in sGFAP (a, b) and sGFAP at V2 or V1 (c, d), or change in sNfL (e, f) and sNfL at V2 or V1 (g, h) with longitudinal brain atrophy lines indicate unadjusted associations derived from linear models between log transformed sGFAP or sNfL, or change in sGFAP or sNfL and PBVC. V0 − V3 indicate the time points between which PBVC was determined, as detailed in Figure 1. The coefficient β with 95% confidence intervals (in brackets) as well as *p*-values are derived from linear models adjusted for age and sex and with an interaction term of MRI parameter and diagnosis. *n*(AQP4-IgG+NMOSD) = 22 and *n*(MOGAD) = 12 for (a, c, e, g); *n*(AQP4-IgG+NMOSD) = 19 and *n*(MOGAD) = 10 for (b, d, f, h). AQP4-IgG+NMOSD, aquaporin 4-immunoglobulin G-seropositive neuromyelitis optica spectrum disorder; MOGAD, myelin oligodendrocyte glycoprotein antibody-associated disease; MRI, magnetic resonance imaging; PBVC, percent brain volume change; sGFAP, serum glial fibrillary acidic protein; sNfL, serum neurofilament light chain; V, visits.

First, we analyzed the association between change in sGFAP and sNfL (V2 − V1) with PBVC. We found no association of change in sGFAP or in sNfL with longitudinal brain atrophy up to V2 (PBVC_V2 − V0_; Figure 3(a) and (e)) in patients with AQP4-IgG+NMOSD or MOGAD. Likewise, no association was detected between change in sGFAP or in sNfL and longitudinal brain atrophy between V1 and V3 (PBVC_V3 − V1_; Figure 3(b) and (f)) in patients with AQP4-IgG+NMOSD or MOGAD.

Next, we investigated the association of sGFAP and sNfL measured at V2 with brain atrophy up to this time point (PBVC_V2 − V0_; Figure 3(c) and (g)). Higher sGFAP at V2 was strongly associated with more extensive longitudinal brain atrophy in patients with AQP4-IgG+NMOSD (β = −0.06 (−0.11, −0.002), *p* = 0.04), while no such association was observed in patients with MOGAD (β = −0.02 (−0.08, 0.05), *p* = 0.55, SES_int_ = 0.2 (−0.3, 0.8), *p*_int_ = 0.37, Figure 3(c)). Similar results were obtained when additionally adjusting for the number of attacks between V0 and V2 (NMOSD: (β = −0.06 (−0.11, −0.0004), *p* = 0.048; MOGAD: β = −0.02 (−0.09, 0.05), *p* = 0.54, SES_int_ = 0.2 (−0.3, 0.8), *p*_int_ = 0.42). sNfL was not associated with prior longitudinal brain atrophy in patients with AQP4-IgG+NMOSD (β = −0.02 (−0.08, 0.03), *p* = 0.38). In patients with MOGAD, higher sNfL was associated with less longitudinal brain atrophy (β = 0.09 (0.02, 0.15), *p* = 0.01, Figure 3(g)).

Lastly, we assessed whether sGFAP and sNfL measured at V1 are prognostic for future longitudinal brain atrophy (PBVC_V3 − V1_; Figure 3(d) and (h)). No associations were observed between sGFAP at V1 and PBVC_V3 − V1_ in patients with AQP4-IgG+NMOSD (β = 0.07 (−0.05, 0.20), *p* = 0.24) or MOGAD (β = 0.02 (−0.19, 0.23), *p* = 0.84, Figure 3(d)). Likewise, sNfL at V1 was not associated with PBVC_V3 − V1_ in AQP4-IgG+NMOSD (β = −0.02 (−0.15, 0.10), *p* = 0.68) or MOGAD (β = 0.06 (−0.14, 0.26), *p* = 0.53, Figure 3(h)).

### Association of sGFAP and sNfL with brain T2-lesion count and volume

#### Cross-sectional analyses

We analyzed the association between sGFAP and sNfL with brain T2-lesion count and volume at baseline. In patients with AQP4-IgG+NMOSD, higher sGFAP was clearly associated with a higher brain T2-lesion volume (β = 1.7 (0.3, 3.2), *p* = 0.02), but not with a higher brain T2-lesion count (β = 0.3 (−0.2, 0.9), *p* = 0.24). In contrast, there was no clear association of higher sGFAP with brain T2-lesion volume (β = 0.5 (−0.2, 1.2), *p* = 0.16) or count (β = 0.6 (−0.6, 1.9), *p* = 0.30) in patients with MOGAD or in HC (volume: β = 4.4 (−5.8, 14.6), *p* = 0.39; count: β = 0.2 (−1.0, 1.3), *p* = 0.78).

No relevant associations were detected between sNfL and brain T2-lesion count in AQP4-IgG+NMOSD (β = −0.3 (−0.8, 0.1), *p* = 0.15), MOGAD (β = 0.1 (−9.8, 1.2), *p* = 0.87), and HC (β = −0.3 (−1.3, 0.7), *p* = 0.55). Likewise, no associations were observed between sNfL and brain T2-lesion volume in AQP4-IgG+NMOSD (β = 0.01 (−1.4, 1.4), *p* = 0.99), MOGAD (β = −0.04 (−0.7, 0.6), *p* = 0.91), and HC (β = −1.8 (−11.2, 7.7), *p* = 0.72).

#### Longitudinal analyses

We also analyzed the association of sGFAP and sNfL with the occurrence of new brain T2-lesions between V1 and V2 in patients with AQP4-IgG+NMOSD. We refrained from analyzing patients with MOGAD and HC, as only three patients with MOGAD showed new T2-lesions and no longitudinal data were available from HC.

**Figure 4.**
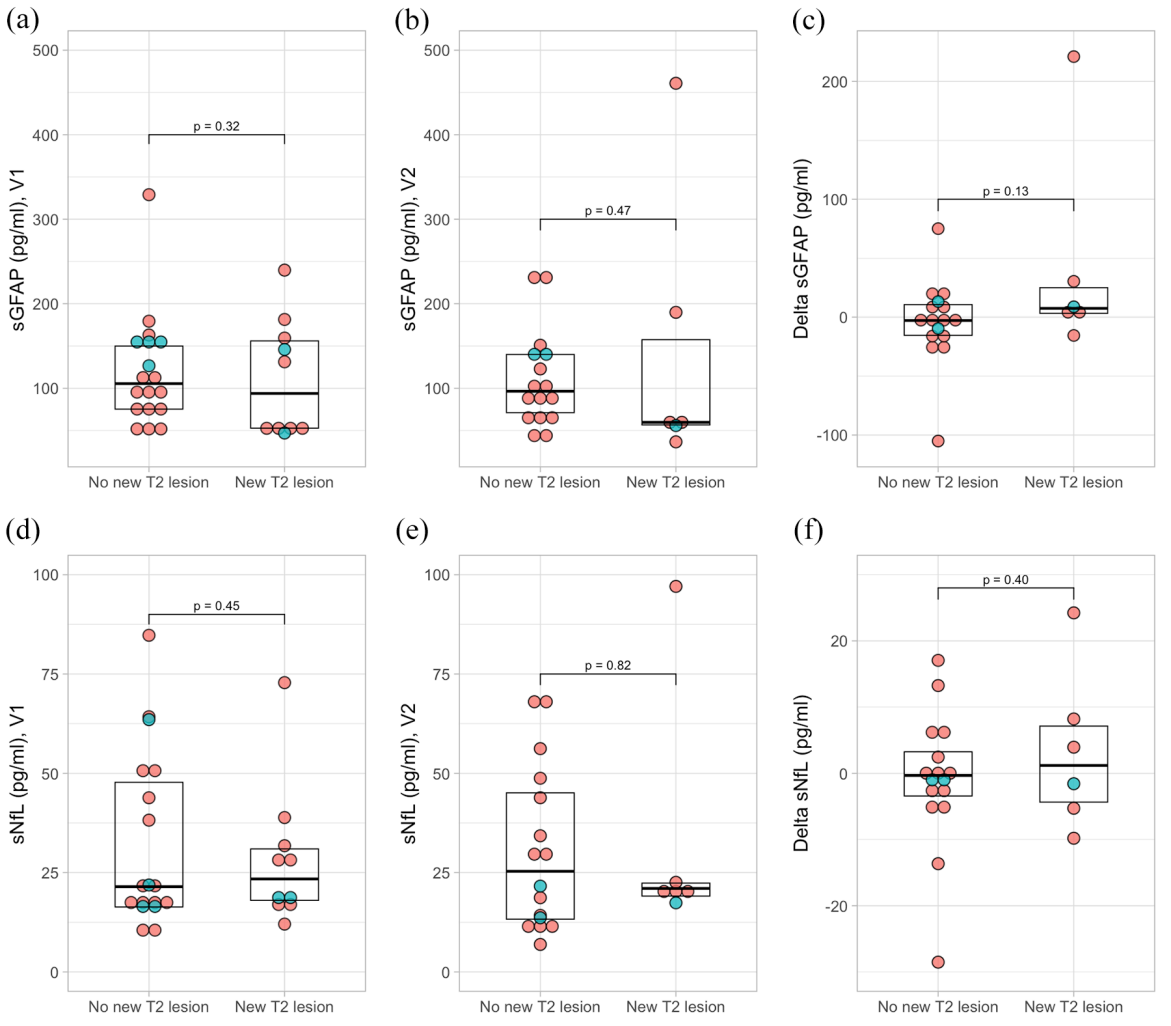
Concentrations and changes in sGFAP (a–c) and sNfL (d–f) in patients with AQP4-IgG+NMOSD with or without new brain MRI T2-lesions. New brain T2-lesion was defined as an increase in the brain T2-lesions count by at least 1 at V2 compared to V1. Delta sGFAP and Delta sNfL indicate the change in sGFAP or sNfL between V2 and V1. Green dots indicate patients with at least one acute attack between V1 and V2, red dots indicate patients without an attack between V1 and V2. *p*-Values are derived from linear models with age, sex, log-transformed sGFAP or sNfL at V1, and new lesion (yes/no) as independent variables. Number of included patients for (a, c) No new T2-lesion, *n* = 18; New T2-lesions, *n* = 10. (b, d) No new T2-lesion, *n* = 16, New T2-lesion, *n* = 6. AQP4-IgG+NMOSD, aquaporin-4-immunoglobulin G-seropositive neuromyelitis optica spectrum disorder; MRI, magnetic resonance imaging; sGFAP, serum glial fibrillary acidic protein; sNfL, serum neurofilament light chain; V, visits.

We grouped patients with AQP4-IgG+NMOSD into those who had or had not at least one new cerebral T2-lesion at V2 compared to V1. Neither sGFAP nor sNfL at V1 nor at V2 differed substantially between patients with or without new brain T2-lesions at V2 (Figure 4). However, median sGFAP between V1 and V2 increased slightly in patients with new T2-lesions (+7 (IQR 3, 25) pg/ml), whereas it decreased slightly in patients without new T2-lesions (−2.8 (−15, 11) pg/ml; *p* = 0.13, Figure 4). sNfL change did not differ between patients with (+1 (−4, 7) pg/ml) and without new T2-lesions (−0.3 (−3, 3) pg/ml; *p* = 0.40; Figure 4). Adjustment for occurrence of an attack between V1 and V2 had no substantial impact on the results (Supplemental Table 1).

## Discussion

In this explorative study, we investigated the associations of sGFAP and sNfL with quantitative brain MRI measures in clinically stable patients with AQP4-IgG+NMOSD as well as MOGAD and HC. The main findings of this investigation are (1) sGFAP is associated with lower thalamus and hippocampus volumes in AQP4-IgG+NMOSD, (2) sGFAP is associated with a higher brain T2-lesion volume in AQP4-IgG+NMOSD, (3) sGFAP is associated with longitudinal brain atrophy in AQP4-IgG+NMOSD. All these findings were either absent or less pronounced in patients with MOGAD and HC, and no similar associations were observed for sNfL.

The selective association of reduced volumes of the thalami and hippocampi, but not other deep gray matter structures, with higher sGFAP in patients with AQP4-IgG+NMOSD is likely to represent a disease-specific phenomenon. Indeed, similar associations were absent in patients with MOGAD and HC. Furthermore, sNfL, which is an unspecific marker of neuroaxonal damage less closely linked to AQP4-IgG+NMOSD pathophysiology than sGFAP, was not associated with thalamic volume in AQP4-IgG+NMOSD. Interestingly, pronounced GFAP expression in morphologically distinct astrocytes has been observed in thalami from general population donors^22^ and thalamic lesions are visible on conventional cerebral MRIs in a proportion of patients with AQP4-IgG+NMOSD.^23^ Together, these findings could indicate a damage of thalamic astrocytes in AQP4-IgG+NMOSD, leading to reduced thalamic volume and elevated sGFAP concentrations. On a functional level, cognitive impairment, which is a frequent symptom in AQP4-IgG+NMOSD, has previously been linked to gray matter, thalamic, and hippocampal volume loss.^8,9^ We are not aware of studies on the association of sGFAP with cognitive impairment in AQP4-IgG+NMOSD, which may be an interesting subject of future research.

The association of sGFAP with brain structural volumes in clinically stable patients with AQP4-IgG+NMOSD implies either (a) that both persistent astrocytopathy and volume loss are sequelae of previous disease activity, that is acute attacks, or (b) that they are related to ongoing subclinical disease activity. The fact that following acute brain damage initially elevated sGFAP levels rapidly decrease^24^ appears consistent with ongoing subclinical disease activity. However, dedicated longitudinal investigations are warranted to further elucidate both possibilities.

We interpret the unexpected association of sGFAP and caudate volume in MOGAD with caution. However, deep gray matter involvement,^17^ including caudate nucleus involvement,^25^ is relatively common in MOGAD and a delayed increase of caudate (as well as thalamic and pallidal) volume has been described in children with MOGAD.^26^ Therefore, further investigation of caudate pathology and its association with fluid biomarkers in independent cohorts appears warranted.

The association of higher sGFAP with higher brain T2-lesion volume in clinically stable patients with AQP4-IgG+NMOSD confirms and extends previous findings. Nonsignificantly higher sGFAP concentrations were previously found in AQP4-IgG+NMOSD patients with versus without brain T2-lesions.^15^ Likewise, CSF GFAP levels,^27^ but not sGFAP levels,^28^ were shown to correlate with spinal cord lesion length. While astrocyte damage and loss as well as secondary neuroaxonal damage are hallmark features of acute lesions in AQP4-IgG+NMOSD, chronic lesions are characterized by astrocytic fibrous gliosis.^29,30^ Such an astrocytic fibrous gliosis might underlie the association of chronic brain T2-lesions with sGFAP, but not sNfL, in AQP4-IgG+NMOSD.

The slight, statistically nonsignificant, sGFAP increase in AQP4-IgG+NMOSD patients with new brain T2-lesions over 1 year would likewise be consistent with astrocytic fibrous gliosis in those lesions. Nevertheless, given the low number of newly occurring brain lesions in this cohort, the observed sGFAP increase should be interpreted cautiously.

The relative specificity of extensive astrocyte damage known to occur in AQP4-IgG+NMOSD, which is much less severe in MOGAD,^31^ might underlie the absence of any association between brain T2-lesions and sGFAP in MOGAD and HC. However, we cannot exclude an influence of a higher number of brain T2-lesions in patients with AQP4-IgG+NMOSD compared to patients with MOGAD and HC on this finding. Nevertheless, the strong association between sGFAP and brain T2-lesions volume underscores the need for a better understanding of “unspecific” brain MRI lesions in AQP4-IgG+NMOSD, whose pathogenesis is currently not completely understood.^32^

The association of longitudinal brain atrophy with sGFAP suggests a possible link between astrocyte damage and subtle neurodegeneration in AQP4-IgG+NMOSD. Interestingly, brain atrophy has recently been recognized in clinically stable patients with AQP4-IgG+NMOSD,^33^ which is not explained by normal aging,^34^ although the extent of this phenomenon remains controversial.^35^ Similarly, subclinical retinal neurodegeneration has been demonstrated in AQP4-IgG+NMOSD.^36,37^ While the mechanisms underlying brain and retinal atrophy in AQP4-IgG+NMOSD have not been fully elucidated, retinal thinning in eyes without a history of optic neuritis has been shown to correlate with higher sGFAP,^13^ supporting a possible relation between ongoing subclinical astrocytic damage and subtle neurodegeneration in AQP4-IgG+NMOSD.

Both sGFAP levels and longitudinal brain atrophy reflect biological processes taking place during a period of time that starts in the past and terminates at the timepoint of interest. The overlap of these periods might underlie the association between sGFAP and past, but not future brain atrophy. Since change in sGFAP reflects the change in the extent of astrocytopathy rather than the extent itself, we speculate that change in sGFAP could correlate with the change in the brain atrophy rate (which could not be assessed in the present investigation) rather than with brain atrophy.

The absence of an association between sGFAP and longitudinal brain atrophy in MOGAD as well as between sNfL and longitudinal brain atrophy in AQP4-IgG+NMOSD suggests a disease-specific process. In several nonprimarily astrocyte-targeting conditions, such as multiple sclerosis^38^ or traumatic brain injury^39^ sNfL is at least as strongly associated with brain atrophy as sGFAP. Speculatively, a recently described extra-lesional sublytic astrocytopathy^30^ in NMOSD might constitute the pathologic correlate of the association between sGFAP and brain atrophy.

This retrospective, monocentric, observational study has several limitations. First, the available cohort size was limited as a result of the low population prevalence of NMOSD and MOGAD. Larger, multicentric studies should thus be conducted to validate the present findings. Second, as we included all patients with available data no sample size calculation was performed. Third, group sizes and demographic metrics, especially age, were unevenly distributed. Fourth, we did not differentiate between “disease-specific” and “unspecific” brain lesions as the identity of “unspecific” brain lesions in NMOSD and MOGAD is subject to current debate. Fifth, intra-individual variability in sGFAP and sNfL could be a potential source for false-positive associations. However, in the present investigation, the intraindividual variability of sGFAP and sNfL was compatible to previous reports.^40,41^ Furthermore, (1) the variability was not different between NMOSD and MOGAD, and (2) was slightly smaller for sNfL than sGFAP while the detected associations were consistently larger for sGFAP, and (3) the group size was smaller for MOGAD than NMOSD, while the detected effects were consistently larger for NMOSD than MOGAD. For these reasons, we assume the impact of intraindividual biomarker variability as a potential confounder is limited in the current investigation.

A strength of this work is the homogenous, well-characterized, longitudinal and clinically stable AQP4-IgG+NMOSD cohort. The inclusion of an HC group and a phenotypically similar, yet pathogenically distinct, disease control group of patients with MOGAD strengthens the specificity of our findings for AQP4-IgG+NMOSD. Finally, brain atrophy was analyzed in a longitudinal fashion, increasing the validity of its association with sGFAP in AQP4-IgG+NMOSD.

## Conclusion

Altogether, we conducted the first systematic evaluation of the association of sGFAP, sNfL, and advanced MRI measures in patients with AQP4-IgG+NMOSD. The findings of this study corroborate sGFAP as a pathophysiology-based biomarker of AQP4-IgG+NMOSD and are compatible with the concept of subclinical disease activity in AQP4-IgG+NMOSD.

## Supplemental Material

sj-docx-1-tan-10.1177_17562864251345792 – Supplemental material for Investigation of the association of serum GFAP and NfL with brain and upper cervical MRI volumes in AQP4-IgG-positive NMOSD and MOGAD Supplemental material, sj-docx-1-tan-10.1177_17562864251345792 for Investigation of the association of serum GFAP and NfL with brain and upper cervical MRI volumes in AQP4-IgG-positive NMOSD and MOGAD by Patrick Schindler, Ulrike Grittner, Rebekka Rust, Susanna Asseyer, Judith Bellmann-Strobl, Tanja Schmitz-Hübsch, Michael Scheel, Sven Jarius, Brigitte Wildemann, Markus Reindl, Pascal Benkert, Jens Kuhle, Friedemann Paul, Klemens Ruprecht and Claudia Chien in Therapeutic Advances in Neurological Disorders

## Declarations

**Ethical approval and consent to participate:** This study was approved by the institutional review board of Charité—Universitätsmedizin Berlin (EA1/041/14, EA1/163/12, and EA1/182/10). All study participants provided written informed consent.

**Consent for publication:** Not applicable.

**Competing interests:** P.S. received speaker honoraria by Roche, received speaker honoraria and travel support by UCB, and received speaker honoraria and served on an advisory board by Alexion. U.G. has nothing to disclose. R.R. received speaking honoraria by Roche unrelated to this study. S.A. has received speaker’s honoraria from Amgen, Alexion, Roche Pharma AG, and Bayer AG. She has received research grants from Novartis, Roche, Berlin Institute of Health and Charité Universitätsmedizin Berlin. J.B.-S. has received institutional research support from NEMOS e.V., Alexion and Bayer AG, a scholarship award from Stiftung Charité (Clinical Fellow Program), personal compensation from Alexion, speaking honoraria and travel grants from Bayer Healthcare, Horizon/Amgen, Novartis and Sanofi-Aventis/Genzyme, in addition received compensation for serving on a scientific advisory board of Alexion, Roche and Merck, all unrelated to the presented work. T.S.-H. received research support by Celgene/bms and speaker’s honoraria by Bayer and Roche, both unrelated to the project. M.S. reports no competing interests. S.J. reports no competing interests. B.W. has received grants from the German Ministry of Education and Research, Deutsche Forschungsgemeinschaft, Dietmar Hopp Stiftung, Klaus Tschira Stiftung, Novartis, Roche, and personal fees from Alexion, INSTAND, Novartis, Roche. M.R. has received a research support from Roche Austria. P.B. has nothing to disclose. J.K. received speaker fees, research support, travel support, and/or served on advisory boards by Swiss MS Society, Swiss National Research Foundation (320030_189140/1), University of Basel, Progressive MS Alliance, Bayer, Biogen, Bristol Myers Squibb, Celgene, Merck, Novartis, Octave Bioscience, Roche, Sanofi. F.P. has received honoraria and research support from Alexion, Bayer, Biogen, Chugai, MerckSerono, Novartis, Genyzme, MedImmune, Shire, and Teva Pharmaceuticals, and serves on scientific advisory boards for Alexion, MedImmune, Novartis, and UCB. He has received funding from Deutsche Forschungsgemeinschaft (DFG Exc 257), Bundesministerium für Bildung und Forschung (Competence Network Multiple Sclerosis), Guthy-Jackson Charitable Foundation, EU Framework Program 7, and National Multiple Sclerosis Society of the USA. He serves on the steering committee of the N- Momentum study with inebilizumab (Horizon Therapeutics) and the OCTiMS Study (Novartis). He is an associate editor with Neurology, Neuroimmunology, and Neuroinflammation and academic editor with PLoS One. K.R. received research support from Novartis, Merck Serono, German Ministry of Education and Research, European Union (821283-2), Stiftung Charité, Guthy-Jackson Charitable Foundation, and Arthur Arnstein Foundation; received speaker’s honoraria from Virion Serion and Novartis. C.C. has received research support from Novartis and Alexion AstraZeneca Rare Disease, is a Standing Committee on Science Member for the Canadian Institutes of Health Research and is a part of a consortium funded by the U.S. Department of Defense; unrelated to this study.

**Availability of data and materials:** The datasets used and analyzed in this study are available from the corresponding author upon reasonable request.
