# Supplementary material for: Investigation of the association of serum GFAP and NfL with brain and upper cervical MRI volumes in AQP4-IgG-positive NMOSD and MOGAD
Source: Ther Adv Neurol Disord. 2025 Jul 20;18:17562864251345792. doi: 10.1177/17562864251345792 (PMC12277671; doi:10.1177/17562864251345792)
Supplement: sj-docx-1-tan-10.1177_17562864251345792 – Supplemental material for Investigation of the association of serum GFAP and NfL with brain and upper cervical MRI volumes in AQP4-IgG-positive NMOSD and MOGAD [file sj-docx-1-tan-10.1177_17562864251345792.docx]

**SUPPLEMENT**

**Investigation of the association of serum GFAP and NfL with brain and upper cervical MRI volumes in AQP4-IgG-positive NMOSD and MOGAD**

**Supplemental Methods**

**1. Participants and study design**

This is an exploratory study. Study participants were recruited at the department of neurology and the experimental and clinical research center of the Charité Universitätsmedizin Berlin. Clinical data as well as serum samples and cerebral MRI scans were obtained from all patients (AQP4-IgG+NMOSD: 33; MOGAD: 17) at the baseline visit (V1). Clinical data included assessment of the expanded disability status scale score (EDSS) by trained raters, current medication, and history of attacks. The baseline visit does not correspond to a specific event in the disease course. From 28 patients with AQP4-IgG+NMOSD and 15 patients with MOGAD, clinical information, serum samples and MRI scans were again obtained at a second visit (V2) a median of 13 [inter quartile range (IQR) 12, 14] and 12 [12, 14] months, respectively, after the baseline visit. Additionally, MRI scans had been obtained at a time point (V0) before the comprehensive baseline visit (V1) from 25 patients with AQP4-IgG+NMOSD (median 28 [26, 31] months before V1) and from 3 patients with MOGAD (median 22 [18, 23] months), applying identical protocols on the same scanner. Furthermore, MRI scans were obtained using identical procedures at a time point (V3) after V2 from 19 patients with AQP4-IgG+NMOSD (median 14 [12, 24] months) and from 11 patients with MOGAD (median 12 [12, 14] months). No further inclusion or exclusion criteria were applied next to participation in the cohort study, diagnosis, and availability at the time of sGFAP and sNfL measurement of serum samples and MRI scans from the respective visit. Serum samples were drawn and MRI scans obtained from all 15 HCs at one time point, applying identical procedures as in the patient groups.

The participants of this study were previously included and reported in studies of sGFAP and sNfL as disease activity and prognostic markers in AQP4-IgG+NMOSD (Schindler et al., 2021), the association of sGFAP and sNfL with OCT parameters (Lin et al., 2022), the longitudinal course of sNfL in MOGAD (Schindler et al., 2024), as well as in studies of advanced cerebral MRI parameters in NMOSD (Chien, Scheel, et al., 2019; Finke et al., 2016; Schmidt et al., 2020)

**2. Methods**

*Laboratory procedures*

Serum AQP4-IgG and MOG-IgG were determined using fixed or live cell-based assays (Euroimmun, Lübeck, Germany; Sven Jarius, University of Heidelberg, Germany; Markus Reindl, Medical University Innsbruck, Austria). None of the patients was AQP4-IgG and MOG-IgG double-seropositive. sGFAP and sNfL were determined by blinded operators using Simoa technology (Quanterix, Lexington, MA, USA) at the Department of Neurology, University of Basel, Switzerland, as previously described (Disanto et al., 2017; Schindler et al., 2021; Watanabe et al., 2019).

*Magnetic resonance imaging*

All cerebral MRI scans were obtained on the same 3-Tesla scanner (Siemens MAGNETOM Trio Tim, Erlangen, Germany), as previously reported (Chien, Oertel, et al., 2019). The MRI protocol included a T1-weighted 3D magnetization-prepared rapid gradient echo and a T2-weighted 3D fluid-attenuated inversion recovery sequence (resolution 1 x 1 x 1 mm each) (Chien, Oertel, et al., 2019). Mean upper cervical cord area (MUCCA) was quantified using JIM7.0 (Xinapse Systems Ltd, UK) software as previously described (Chien et al., 2018), and brain lesion segmentation was manually performed as previously reported (Lin et al., 2024).

*Statistical analyses*

Given the skewed distribution of sGFAP and sNfL data, log-transformation (log10(GFAP-25, log10(NfL-3.5)) was performed to achieve more symmetric distributions. The constants (-25 and -3.5, respectively) were empirically determined to optimize distributional symmetry. We validated the transformation by examining normality plots, skewness statistics, and model residuals. Standardized mean differences (SMD) were calculated using package “tableone” (Pollard et al., 2018) for categorical and sufficiently normally distributed, and “TOSTER” (Lakens, 2017) for non-normally distributed variables. For descriptive group comparison of baseline characteristics, package “tableone” was used. Marginal effect estimates beta (β) were calculated using package “emmeans” (Lenth, 2022). Standardized effect sizes (SES) were calculated using package “effectsize” (Ben-Shachar MS, 2020). All statistical analyses were conducted and all figures created using R version 4.2.2. Further R packages used were “tidyverse”, “lubridate”, and “patchwork” (Wickham H & E, 2019).

**Supplemental Figures**


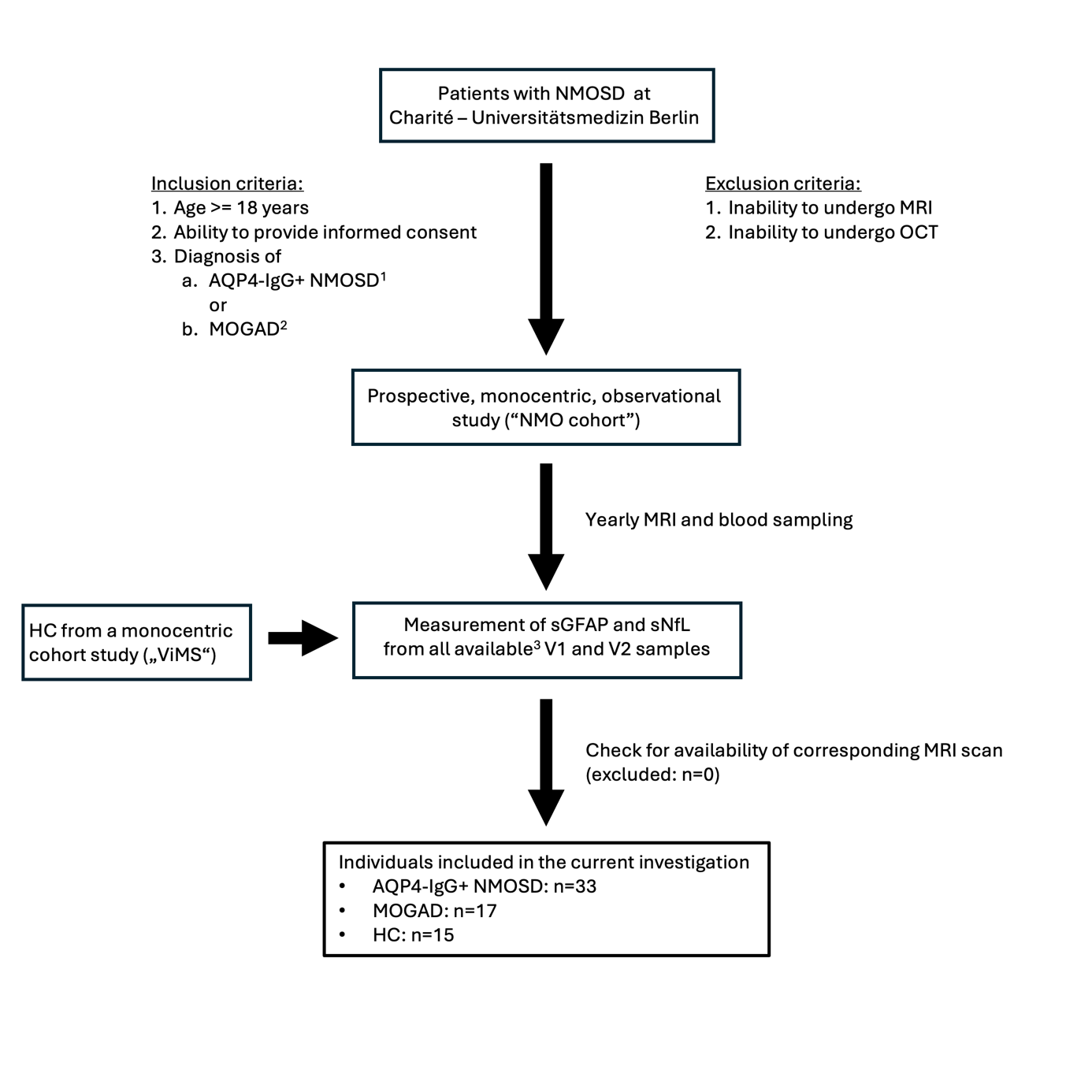


**Supplemental Figure 1: Study participants.**

1: According to the 2015 IPND criteria (Wingerchuk et al. Neurology 2015); 2: according to the diagnostic criteria proposed by Jarius et al. (J Neuroinflam 2018) or Banwell et al. (Lancet Neurol 2023); 3: available at the the time of measurement in 2018.

Abbreviations: AQP4-IgG+NMOSD = aquaporin-4-immonuglobulin G seropositive neuromyelitis optica spectrum disorder; HC = healthy controls; MOGAD = Myelin oligodendrocyte glycoprotein associated disease; MRI = magnetic rsonance imaging; OCT = optical coherence tomography; sGFAP= serum glial fibrillary acidic protein; sNfL = serum neurofilament light chain


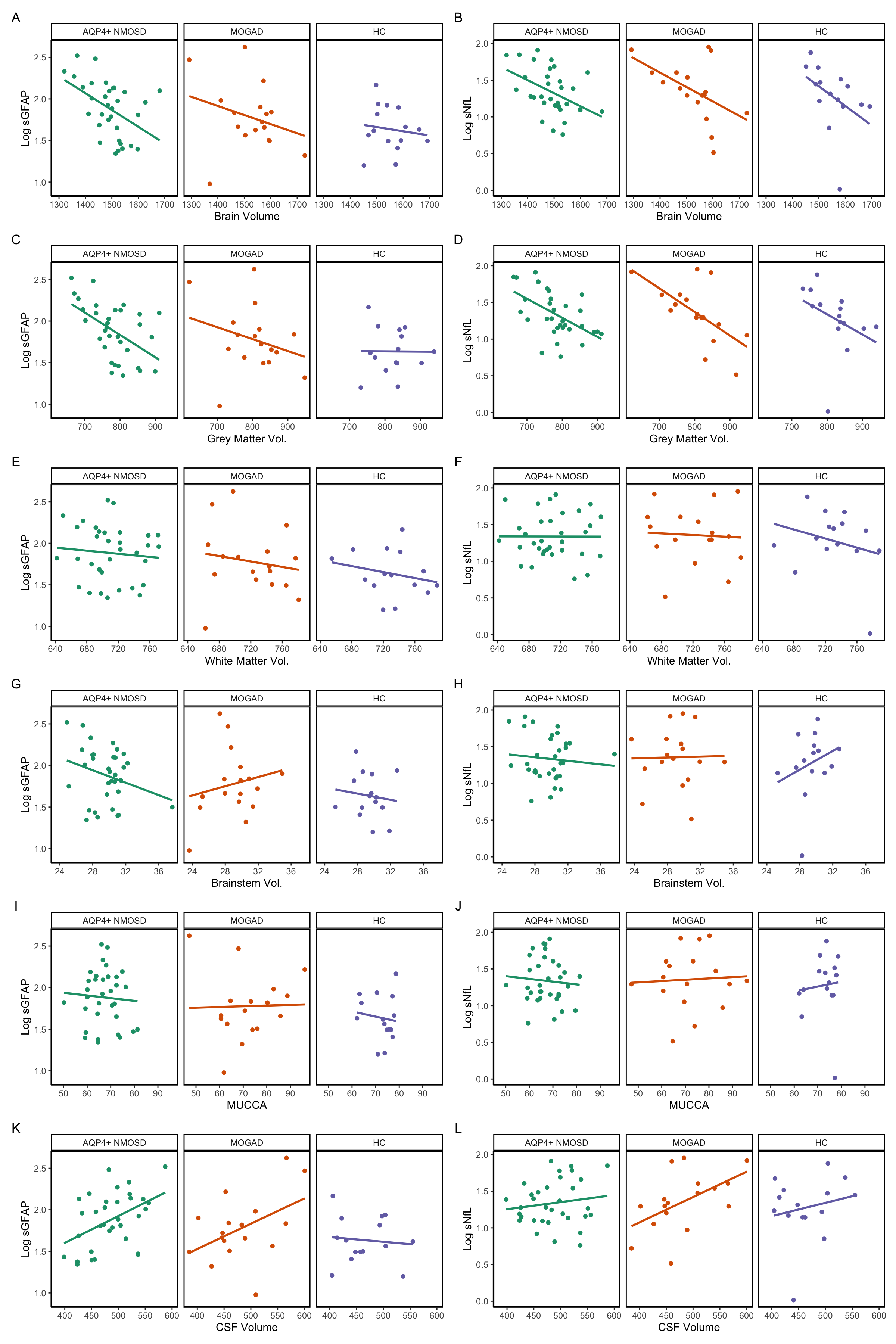


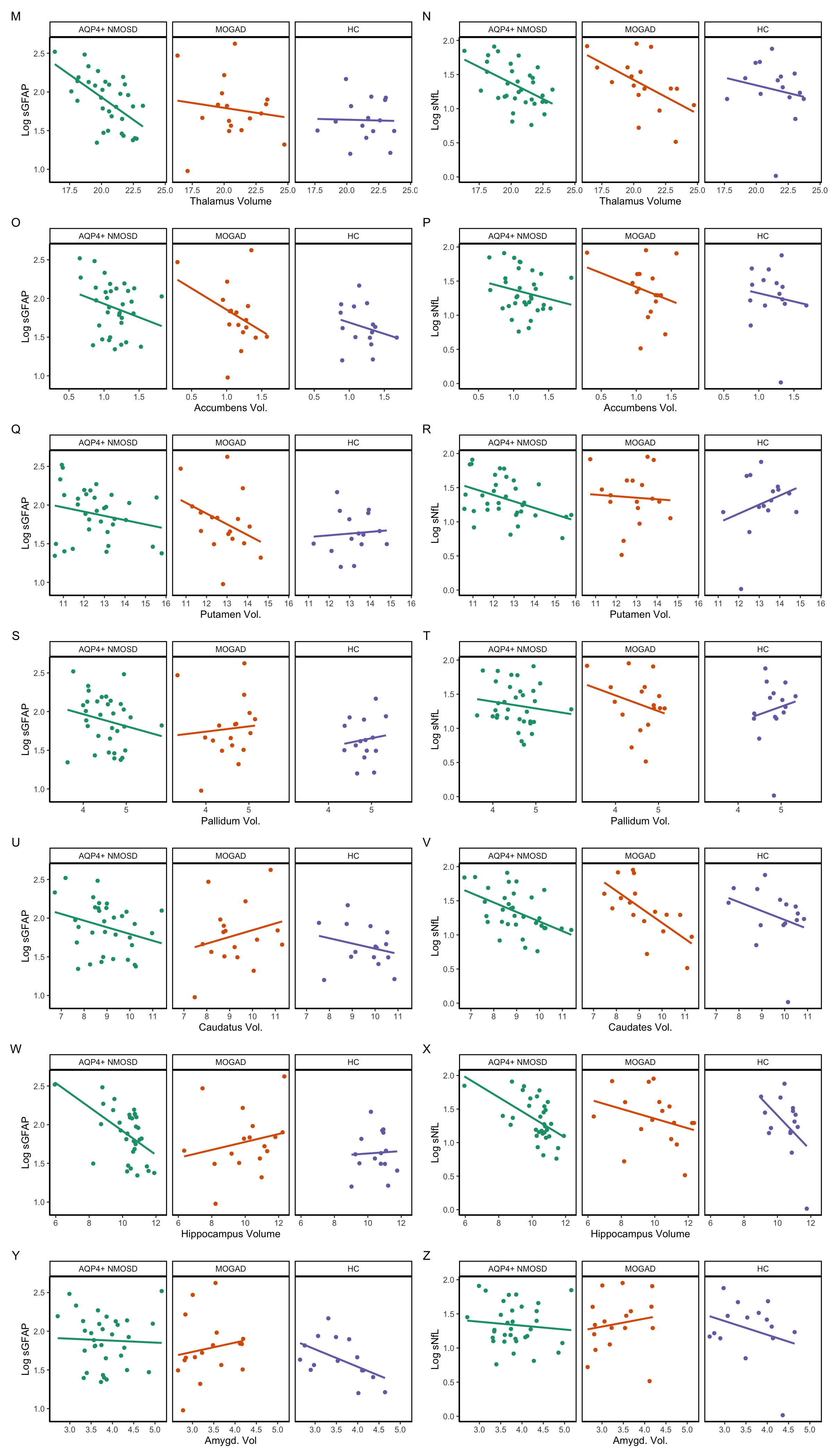


**Supplemental Figure 2: Baseline associations between sGFAP (A) or sNfL (B) and brain MRI structural volumes.**

Lines indicate unadjusted associations derived from linear models between log transformed sGFAP or sNfL and the respective MRI parameter.

Abbreviations: AQP4+ NMOSD = aquaporin 4-IgG seropositive neuromyelitis optica spectrum disorder, HC = healthy controls, MOGAD = myelin oligodendrocyte glycoprotein associated disease, sGFAP = serum glial fibrillary acidic protein, sNfL = serum neurofilament light chain

**Supplemental Tables**

**Supplemental Table 1: Difference in sGFAP, sNfL, and longitudinal change in sGFAP and sNfL in patients with AQP4-IgG+NMOSD with or without new brain MRI T2-lesions.**

| **No adjustment for attack between V1 & V2** | | | | | | | | | |
| --- | --- | --- | --- | --- | --- | --- | --- | --- | --- |
|  | **V1** | | | **V2** | | | **Delta V2-V1** | | |
|  | *beta* | *95%-CI* | *p* | *beta* | *95%-CI* | *p* | *beta* | *95%-CI* | *p* |
| **sGFAP** | 0.11 | -0.11 – 0.34 | 0.32 | 0.11 | -0.21 – 0.44 | 0.47 | -4.7 | -11 - 1.5 | 0.13 |
| **sNfL** | 0.05 | -0.09 – 0.20 | 0.45 | -0.02 | -0.23 – 0.19 | 0.82 | -2.1 | -7.3 – 3.1 | 0.40 |
| **Adjustment for attack between V1 & V2** | | | | | | | | | |
|  | **V1** | | | **V2** | | | **Delta V2-V1** | | |
|  | *beta* | *95%-CI* | *p* | *beta* | *95%-CI* | *p* | *beta* | *95%-CI* | *p* |
| **sGFAP** | 0.11 | -0.11 – 0.33 | 0.31 | 0.13 | -0.19 – 0.45 | 0.41 | -4.6 | -11 – 1.8 | 0.15 |
| **sNfL** | 0.05 | -0.09 – 0.20 | 0.46 | -0.02 | -0.24 – 0.19 | 0.82 | -2.1 | -7.5 – 3.2 | 0.40 |

“New brain T2 lesion” indicates an increase in the brain T2-lesions count by at least 1 at V2 compared to V1. Delta sGFAP and Delta sNfL indicate the change in sGFAP or sNfL between V2 and V1.

Statistical analysis: The difference in sGFAP and sNfL between patients with or without new T2-lesions was analyzed by linear models with log-transformed “sGFAP” or “sNfL” as dependent variable and “age”, “sex”, and “New T2-lesion yes / no” as independent variables. The difference in change of sGFAP or sNfL was analyzed by linear models with rank transformed “sGFAP_V2-V1” or “sNfL_V2-V1” as dependent variable and “age”, “sex”, “log transformed sGFAP or sNfL at V1” and “New T2-lesion yes / no” as independent variables. Adjustment for occurrence of an attack between V1 and V2 was conducted by inclusion of “attack between V1 and V2 yes / no” as an independent categorical variable.

Number of included patients for A, C: No new T2-lesion, n = 18; New T2-lesions, n = 10. B, D: No new T2-lesion, n = 16, New T2-lesion, n = 6.

Abbreviations: sGFAP = serum glial fibrillary acidic protein, sNfL = serum neurofilament light chain, V1 = visit 1, V2 = visit 2.

**References**

Ben-Shachar MS, L. D., Makowski D. (2020). effectsize: Estimation of Effect Size Indices and Standardized Parameters. *Journal of Open Source Software*, *5*, 2815. <https://doi.org/doi:10.21105/joss.02815>

Chien, C., Brandt, A. U., Schmidt, F., Bellmann-Strobl, J., Ruprecht, K., Paul, F., & Scheel, M. (2018). MRI-Based Methods for Spinal Cord Atrophy Evaluation: A Comparison of Cervical Cord Cross-Sectional Area, Cervical Cord Volume, and Full Spinal Cord Volume in Patients with Aquaporin-4 Antibody Seropositive Neuromyelitis Optica Spectrum Disorders. *AJNR Am J Neuroradiol*, *39*(7), 1362-1368. <https://doi.org/10.3174/ajnr.A5665>

Chien, C., Oertel, F. C., Siebert, N., Zimmermann, H., Asseyer, S., Kuchling, J., Scheel, M., Ruprecht, K., Bellmann-Strobl, J., Paul, F., & Brandt, A. U. (2019). Imaging markers of disability in aquaporin-4 immunoglobulin G seropositive neuromyelitis optica: a graph theory study. *Brain Commun*, *1*(1), fcz026. <https://doi.org/10.1093/braincomms/fcz026>

Chien, C., Scheel, M., Schmitz-Hübsch, T., Borisow, N., Ruprecht, K., Bellmann-Strobl, J., Paul, F., & Brandt, A. U. (2019). Spinal cord lesions and atrophy in NMOSD with AQP4-IgG and MOG-IgG associated autoimmunity. *Mult Scler*, *25*(14), 1926-1936. <https://doi.org/10.1177/1352458518815596>

Disanto, G., Barro, C., Benkert, P., Naegelin, Y., Schadelin, S., Giardiello, A., Zecca, C., Blennow, K., Zetterberg, H., Leppert, D., Kappos, L., Gobbi, C., & Kuhle, J. (2017). Serum Neurofilament light: A biomarker of neuronal damage in multiple sclerosis. *Ann Neurol*, *81*(6), 857-870. <https://doi.org/10.1002/ana.24954>

Finke, C., Heine, J., Pache, F., Lacheta, A., Borisow, N., Kuchling, J., Bellmann-Strobl, J., Ruprecht, K., Brandt, A. U., & Paul, F. (2016). Normal volumes and microstructural integrity of deep gray matter structures in AQP4+ NMOSD. *Neurol Neuroimmunol Neuroinflamm*, *3*(3), e229. <https://doi.org/10.1212/nxi.0000000000000229>

Lakens, D. (2017). Equivalence tests: A practical primer for t-tests, correlations, and meta-analyses. *Social Psychological and Personality Science*, *1*. <https://doi.org/10.1177/1948550617697177>

Lenth, R. (2022). *emmeans: Estimated Marginal Means, aka Least-Squares Means_. R package version 1.8.0*. In <[https://cran.r-project.org/package=emmeans](https://CRAN.R-project.org/package=emmeans)>

Lin, T.-Y., Schindler, P., Grittner, U., Oertel, F. C., Lu, A., Motamedi, S., Yadav, S. K., Duchow, A. S., Jarius, S., Kuhle, J., Benkert, P., Brandt, A. U., Bellmann-Strobl, J., Schmitz-Hübsch, T., Paul, F., Ruprecht, K., & Zimmermann, H. G. (2022). Serum glial fibrillary acidic protein correlates with retinal structural damage in aquaporin-4 antibody positive neuromyelitis optica spectrum disorder. *Mult Scler Relat Disord*, 104100. <https://doi.org/https://doi.org/10.1016/j.msard.2022.104100>

Lin, T. Y., Chien, C., Kuchling, J., Asseyer, S., Motamedi, S., Bellmann-Strobl, J., Schmitz-Hübsch, T., Ruprecht, K., Brandt, A. U., Zimmermann, H. G., & Paul, F. (2024). Interactions of optic radiation lesions with retinal and brain atrophy in early multiple sclerosis. *Ann Clin Transl Neurol*, *11*(1), 45-56. <https://doi.org/10.1002/acn3.51931>

Patenaude, B., Smith, S. M., Kennedy, D. N., & Jenkinson, M. (2011). A Bayesian model of shape and appearance for subcortical brain segmentation. *Neuroimage*, *56*(3), 907-922. <https://doi.org/10.1016/j.neuroimage.2011.02.046>

Pollard, T. J., Johnson, A. E. W., Raffa, J. D., & Mark, R. G. (2018). tableone: An open source Python package for producing summary statistics for research papers. *JAMIA Open*, *1*(1), 26-31. <https://doi.org/10.1093/jamiaopen/ooy012>

Schindler, P., Bellmann-Strobl, J., Kuhle, J., Wildemann, B., Jarius, S., Paul, F., & Ruprecht, K. (2024). Longitudinal change of serum NfL as disease activity biomarker candidate in MOGAD: A descriptive cohort study. *Mult Scler Relat Disord*, *88*. <https://doi.org/10.1016/j.msard.2024.105729>

Schindler, P., Grittner, U., Oechtering, J., Leppert, D., Siebert, N., Duchow, A. S., Oertel, F. C., Asseyer, S., Kuchling, J., Zimmermann, H. G., Brandt, A. U., Benkert, P., Reindl, M., Jarius, S., Paul, F., Bellmann-Strobl, J., Kuhle, J., & Ruprecht, K. (2021). Serum GFAP and NfL as disease severity and prognostic biomarkers in patients with aquaporin-4 antibody-positive neuromyelitis optica spectrum disorder. *J Neuroinflammation*, *18*(1), 105. <https://doi.org/10.1186/s12974-021-02138-7>

Schmidt, F. A., Chien, C., Kuchling, J., Bellmann-Strobl, J., Ruprecht, K., Siebert, N., Asseyer, S., Jarius, S., Brandt, A. U., Scheel, M., & Paul, F. (2020). Differences in Advanced Magnetic Resonance Imaging in MOG-IgG and AQP4-IgG Seropositive Neuromyelitis Optica Spectrum Disorders: A Comparative Study. *Front Neurol*, *11*, 499910. <https://doi.org/10.3389/fneur.2020.499910>

Smith, S. M., Zhang, Y., Jenkinson, M., Chen, J., Matthews, P. M., Federico, A., & De Stefano, N. (2002). Accurate, robust, and automated longitudinal and cross-sectional brain change analysis. *Neuroimage*, *17*(1), 479-489. <https://doi.org/10.1006/nimg.2002.1040>

Watanabe, M., Nakamura, Y., Michalak, Z., Isobe, N., Barro, C., Leppert, D., Matsushita, T., Hayashi, F., Yamasaki, R., Kuhle, J., & Kira, J. I. (2019). Serum GFAP and neurofilament light as biomarkers of disease activity and disability in NMOSD. *Neurology*, *93*(13), e1299-e1311. <https://doi.org/10.1212/wnl.0000000000008160>

Wickham H, A. M., Bryan J, Chang W, McGowan LD, François R, Grolemund G, Hayes A, Henry L, Hester J, Kuhn M, Pedersen TL, Miller, & E, B. S., Müller K, Ooms J, Robinson D, Seidel DP, Spinu V, Takahashi K, Vaughan D, Wilke C, Woo K, Yutani H. (2019). Welcome to the tidyverse. *Journal of Open Source Software*. <https://doi.org/10.21105/joss.01686>
